# An Experiment That Failed

**Published:** 1919-11

**Authors:** C. N. Johnson


### AN EXPERIMENT THAT FAILED

BY C. N. JOHNSON, D.D.S.

This is a wholly personal epistle. It need not, therefore, be read by those who care little for the personal experiences of others, and I imagine that most people are in this category. I was to take a trip from Chicago to the great Canadian Northwest to attend some dental meetings in Saskatchewan and Alberta, and the prospect which under ordinary circumstances would have delighted me beyond measure, left me in the deepest depression. It was not that I did not look forward with the most pleasant anticipation to seeing my good friends of the prairie provinces, nor did I fail to appreciate the honor of an invitation to meet with them. But I was to make the journey alone, and this was what depressed me. It was the first long trip I had ever taken without having my family with me for company, and the prospect was not attractive.

So for diversion I resolved on an experiment. Travelling alone as I was, I conceived the brilliant idea of pretending that I had committed some terrible crime, and then seeing how successfully I could escape. I had heard of criminals getting away and leaving no trace, and I figured that I could hide my identity as skillfully as most criminals. The great difficulty was to select the proper crime. I first thought of murder, but in Chicago this crime had become so common that there was no class to it. I wanted to do the thing up with distinction, and when I recalled the fact that there had been more than thirty murders committed in Chicago in a few days during the race riots, I concluded that this particular crime had been somewhat overdone. Then I thought of the hold-up and robbery, but this also had become so commonplace and everyday in character that there was no originality about it. Robbing a bank did appeal to me for a brief moment till I counted the number of cases around Chicago in recent weeks, and had to acknowledge that I was outclassed. Once started out on the enterprise, I was determined that my crime should be well worth while, and I accordingly canvassed the whole category, only to be confronted with the humiliating fact that none of them seemed really unique or distinctive. Suddenly, I had an inspiration. I hit upon the most marvelous and heinous crime of all, and what was better, it was one decidedly dental. I would fill a pulpless tooth for a patient, and actually leave it in the mouth without cutting down through the jaw and dissecting out a piece for microscopical examination to count the number of death-dealing micro-organisms found lodged at the apex of the root. This, I figured, after reading certain articles on the subject and hearing the dicta of certain of our medical friends, was the very acme of all criminal procedures, and I was so obsessed with the desire to pose as a criminal that I determined to do this desperate thing. And so I left Chicago with the cloud of this terrible crime hanging over me, fully resolved that I was going to travel incognito, and see how far up the line I could get without having my identity revealed. It was a pleasing little experiment, and I entered into it with great enthusiasm.

The game was to start the moment I entered the railway depot to take my train. I had just said good-bye to the dear ones who came to see me off, and turned to lose my identity in the great moving mass of humanity who surged toward the gates, when I was suddenly greeted with, "Hello, Doctor, which train are you going on?" And my suitcase was grabbed from my hand, and the way led to the private gate, away from the crowd. It was one of my boys from the college, who was working at the station during vacation—and let it be said parenthetically that this is the sort of boy who usually makes good—the one who works his way through college. The moment I was in the train yard I looked across the track and saw my good old friend, Mr. Healy—"Dan" Healy—the very greatest dining-car conductor in the world—bar none. He has had charge of the dining-car on the Pioneer Limited out of Chicago for more than thirty years, and if there is a single individual among all the travelling public who does not know Mr. Healy, I would like to see this individual and ask him where he has been buried for the past thirty years. Mr. Healy is the one original genius in his line. He looks after his "guests" with the most solicitous care, and he has done more to make the Pioneer Limited popular than any other one thing. When he saw me, he said: "Doctor, let the boy put your suitcase in the sleeper, and you come with me." The diner is always filled instantly after the train starts, so Mr. Healy took me down the track and when the train backed in we got directly aboard the diner and I was soon seated comfortably. We chatted nearly all the way to Milwaukee, or as often as he could get away from his "guests," and he fed me almost to suffocation. But it all digested well, because it was savored with the greatest good will and the most solicitous care on Mr. Healy's part. Long live Dan Healy—he is the salt of the earth.

Plainly, I was not getting on well with my experiment. But I said to myself that after I left Milwaukee I was safe, and so I went to bed gloating over my crime and secure from detection. The next morning as I stepped off the train at St. Paul, the first man I ran into was Dr.

E. K. Wedelstaedt, who bundled me into his car and drove me to his home—just like being arrested, you know. I put in a most delightful day with Dr. and Mrs. Wedelstaedt, and forgot all about my crime. In the afternoon they took me to Minneapolis in their car, and I called on Elmer Best and Fred Kremer. Neither one treated me as if I were a criminal. I was feeling low-spirited at my failure so far, but consoled myself with the reflection that after I left Minneapolis all would be well. As the train pulled out for Winnipeg, I lay back in my seat and reveled in the certainty that not a soul on board knew me and not one was conscious of the crime I had committed. At last I was making headway with my experiment, and—"Well, I declare, if here isn't Dr. Johnson!" I looked up guiltily and saw standing over me a gentleman and a very sweet-faced girl—Dr. A. M. Harrison and his daughter, of Rockford, Illinois. They were on their way up to the Canadian Rockies, and fate framed it that they should be on the same train with me. But I am sure they never suspected me of being an escaped criminal. The romance was oozing out of my experiment, but the pleasure was creeping in.

At Winnipeg, I left the train to take a taxi from one station to another. "Hello, Doctor; what kind of a trip did you have?" It was Dr. Taylor—Dr. Wm. F. Taylor—or rather "Bill" Taylor, as he is affectionately called by his friends. I did not dream that Dr. Taylor knew I was still in the land of the living, let alone in the land of the Maple Leaf. He "had heard" that I was passing through, and thought he "would drop around to the station." He put me in his car, and we lived in that car all day long, with intervals of calling on friends and eating. No one would ever have imagined that Bill Taylor had an office that whole day. I saw Winnipeg and its environs as I had never seen them before. It almost seemed as if William had learned of my crime and was determined that I should not escape. It was the most pleasant detention I

had ever experienced, and I figured that being a criminal was not so bad after all.

We called on many of the Winnipeg men and visited the clinics of the C.A.D.C. We saw among others, Drs. Clint—the elder Clint is easily the youngest man of his years in the profession—Greenfield, J. F. Taylor, Bannerman, and others whose names I do not recall—all splendid men. The spirit of the West is typified by these men of Winnipeg and it is a spirit to be reckoned with. It is the spirit that put down lawlessness and restored order in the recent strike. No one will ever know the heroism that was displayed by the citizens of Winnipeg at that time. These men, who were today showing me around Winnipeg as if nothing had happened, were at the time of the strike out of their offices for five weeks, doing duty for the city. A citizens' committee was organized to make Winnipeg safe and sane. Of course Bill Taylor was in it. Bert Greenfield was a member of the improvised fire department—along with some of the best citizens of Winnipeg. "And a mighty good fire department we had," said Bert with a laugh. They delivered ice to the needy, milk to the babies, and looked after the comfort and welfare of the helpless in every way. Men turned over their finest automobiles to the committee for delivery wagons. One new Packard car was offered by its owner to take ice to the housewives —the only stipulation being that the men refrain from chopping the ice on the floor of the tonneau.

Is it any wonder that men of this spirit finally restored order in the face of the meanest foreign raid that was ever made on the rights of responsible government? Don't worry about Winnipeg. Her citizens are alert and no such nonsense will ever be tolerated again.

Dr. Bannerman took me to his office and showed me some of the finest furs I have ever seen. He is a sportsman of the highest type—by nature, by instinct, and by opportunity. It is invigorating to talk with him. He slipped in my pocket a pair of the prettiest moccasins a man—or a woman—ever wore. He also introduced me to a type of man I always wanted to meet—a man in the employ of the Hudson Bay Company, who lives away up a few miles this side of the North Pole. His name was Mr. Flett—Alex Flett—and he is one of the noblemen of the North. It was the first time in four years that he had been out into civilization, and it was a rare treat to talk to him. He was quiet, unobtrusive, smiling, and soft of speech, as if the mysteries of the Great North Land had subdued him into a gentleness that was almost sublime. And yet there was that indefinable reserve force about the man that made one feel instinctively that in the emergencies and dangers of the North one would need no better protector than he. It was refreshing, in the tempest and turmoil of present day unrest, to meet a man over whom the unsettled tendencies of the age broke with so little effect, and who seemed superior to the smaller things of life. I found myself longing with an intensity which can never be appeased for an extended journey into the wildernesses of the earth, where I could remain secure from the clamor of modern life; where I could study nature at first hand and commune with the wild things of the forest and stream; where I could forget for the time the piercing scream of the steam whistle or the harsh crashing of an elevated train; where I could look up at the stars at night and listen to the sounds of the beaver building his dam; where time is not counted by the seconds or the minutes, but by the revolution of the spheres; where—but hold—this is a vain dream which I must indulge no longer. I am a hothouse plant, spoiled and unseasoned by the cruel years of artificial life, and I must content myself to live out my allotted span minus a breath of that real life which is the daily portion of our good friend Alex Flett.

Such a day as I had in Winnipeg, and all because of the big-heartedness of Dr. Taylor! I am sorry for anyone in the profession who has not met Bill Taylor. He is one of the princes of the earth. When the names of those who have lived for their fellow-men are written on the scroll, the name of Wm. F. Taylor will be well up on the list, and I can imagine that when Bill sees that he will get a ladder and begin to erase it, and then I can also imagine that the Recording Angel will wave William back with a gentle smile and write his name in larger letters than before.

Some one has said that the real art of writing is to know when to stop. I could go on writing of Winnipeg and her citizens interminably, but the limits of space prevent.

I think all will admit that my experiment failed, and I am unregenerate enough to be pleased that it did. In the next issue I hope to have something to say of the dentists of Saskatchewan and Alberta.

NOTE.—This is a letter written by Dr. C. N. Johnson to *Oral Health,* and it is so characteristic of the man, and is so fine an expression of the way one would like to feel towards his fellow-men, that we feel it ought to be reprinted and read by all people who are in these troublesome times inclined to believe that all men have lost not only reason, but the natural kindly feelings through which, if everyone allowed them to control his activities, as well as his sentiments, there would be a quieting of the turbulence of the great world's unrest.—Editor *Register.*

